# Predicting Nomogram for Severe Oral Mucositis in Patients with Nasopharyngeal Carcinoma during Intensity-Modulated Radiation Therapy: A Retrospective Cohort Study

**DOI:** 10.3390/curroncol30010017

**Published:** 2022-12-23

**Authors:** Zhibing Liu, Lulu Huang, Housheng Wang, Zhiling Shi, Yaqin Huang, Lixing Liang, Rensheng Wang, Kai Hu

**Affiliations:** 1 Department of Radiation Oncology, The First Affiliated Hospital of Guangxi Medical University, Nanning 530021, China; 2 Laboratory of Early Prevention and Treatment for Regional High Frequency Tumor (Guangxi Medical University), Ministry of Education, Nanning 530021, China; 3 Guangxi Key Laboratory of Immunology and Metabolism for Liver Diseases, Nanning 530021, China

**Keywords:** nasopharyngeal carcinoma, nomograms, stomatitis, intensity-modulated, radiotherapy

## Abstract

Background: Oral mucositis is an acute adverse reaction with high incidence during radiotherapy. Severe oral mucositis can seriously affect patients’ quality of life and compliance with radiotherapy. The aim of this study was to identify the risk factors for severe oral mucositis and to develop a nomogram for predicting severe oral mucositis in patients with nasopharyngeal carcinoma. Methods: One hundred and ninety patients with nasopharyngeal carcinoma were retrospectively screened in this study. Least absolute shrinkage and selection operator regression and multivariate logistic regression analyses were performed to identify the best predictors of severe oral mucositis. A nomogram was constructed based on the factors. Finally, the discriminative ability of the nomogram was evaluated. Results: Four independent factors predicting severe oral mucositis were identified: age, N stage, the cycle of induction chemotherapy, and dose-volumetric parameter V40 (%) of oral cavity. The area under the receiver of operating characteristic curve of the nomogram was 0.759 (95% confidence interval: 0.691–0.827). Conclusions: A predictive nomogram for severe oral mucositis was established and validated in this study. The nomogram provides a reliable and practical model for clinically predicting the probability of severe oral mucositis in patients with nasopharyngeal carcinoma before intensity-modulated radiation therapy.

## 1. Introduction

There were 129,000 new cases of Nasopharyngeal Carcinoma (NPC) worldwide in 2018, accounting for 0.7% of all cancers [1]. Although the incidence is not very high, the distribution of NPC patients is unbalanced, tending to occur in southern China, especially in Guangdong and Guangxi provinces [2]. In agreement with the complex anatomical structure of the nasopharynx and NPC’s high sensitivity to radiation, radiotherapy is the most important treatment for NPC [3]. Oral Mucositis (OM) is one of the most common and serious adverse events in patients with NPC who receive radiotherapy. Clinical manifestations of OM include oral mucosal congestion, erythema, pain, difficulty in eating, and alterations in taste sensation (dysgeusia) [4,5,6,7]. There are several assessment scales for grading the severity of oral mucositis currently, including the Common Toxicity Criteria (CTC version 4.0), the Toxicity criteria of the Radiation Therapy Oncology Group (RTOG), the European Organization for Research and Treatment of Cancer (EORTC), and the criteria set out by the World Health Organization (WHO) and the OM Assessment Scale (OMAS) [8,9]. Among the above-mentioned grading standards, the WHO oral toxicity scale can combine changes in mucosal morphology with dysfunction and has been widely used. Patients with Severe Oral Mucositis (SOM), which is defined as grade 3–4 OM by the WHO [10], are more susceptible to secondary infections of oral mucosa or even widespread systemic inflammatory response [11] that may lead to interruption of radiotherapy. Intensity-Modulated Radiotherapy (IMRT) has become the most commonly used radiation treatment technique for localized NPC [12]. However, randomized controlled trials have shown that IMRT did not reduce the incidence of OM in patients with NPC. More than 80% of these patients presented with OM during irradiation; in particular, about 50% of patients developed SOM [13]. Therefore, early identification of individuals at high risk for SOM is essential for disease prevention and intervention so that measures can be taken to reduce the incidence of SOM, thus improving therapy against NPC.

It is unclear which factors can be used clinically to provide individualized risk prediction for SOM. Li et al. [14] found that dose-volumetric parameters V30 and V50 of the oral cavity were independent predictors of SOM in patients with NPC receiving IMRT. However, the study was conducted six years ago, when the treatment paradigm differed considerably from the current treatment paradigm, such as the use of immunotherapy. The influence of clinical and dosimetric parameters of radiotherapy, including age; immunotherapy, nimotuzumab, or chemotherapy; sex; diabetes; T stage; and N stage, which may affect the occurrence of SOM, also needs to be evaluated. Given the serious adverse effects of SOM, we believe that it is very important to identify the susceptibility factors of SOM to enable early intervention. However, to date, valid prediction models for SOM in patients with NPC treated with IMRT are lacking. We hypothesize that dose-volume parameters and clinical variables be predictors of individual patient risk for developing SOM. Thus, the present study aimed to identify individual-level risk factors of SOM in patients with NPC who received IMRT and to develop a nomogram to predict SOM.

## 2. Materials and Methods

### 2.1. Patients

This study was performed at the First Affiliated Hospital of Guangxi Medical University. We performed a systematic and comprehensive analysis of clinical variables and radiation dosimetry parameters in patients with pathologically confirmed NPC who underwent evaluation and treatment between June 2021 and March 2022.

The inclusion criteria for this study were as follows: (1) having newly diagnosed, pathologically confirmed, untreated NPC, WHO type II or III; (2) having stage I–IVa according to the 8th edition of the Union for International Cancer Control; (3) having completed the entire process of radical IMRT and obtained complete treatment plan data; and (4) having no oral disease and no history of oral surgery. The exclusion criteria for this study were as follows: (1) palliative radiotherapy; (2) history or presence of other malignant tumors; (3) pregnancy or lactation; and (4) co-existence of other major diseases, including heart failure, severe hepatitis, or renal dysfunction.

This study was approved by the Ethics Committee of the First Affiliated Hospital of Guangxi Medical University (No. 2022-E455-01, 15 January 2022). Informed consent was obtained from all the patients.

### 2.2. Treatment

#### 2.2.1. Radiotherapy

The formulation of radiotherapy planning follows the International Commission on Radiation Units and Measurements Report 62 guidelines and Chinese guidelines for radiotherapy for nasopharyngeal carcinoma [15]. All patients received radical IMRT. The NPC irradiation target volume includes the Gross Tumor Volume (GTVnx), Gross Tumor Volume involving lymph nodes (GTVnd), and Clinical Target Volume (CTV). The primary tumor target volume was delineated based on magnetic resonance imaging, computed tomography, and positron emission tomography/CT. Depending on local progress, the delineation of the CTV covers higher-, intermediate-, or lower-risk locations. GTVnx refers to the volume of a gross nasopharyngeal tumor observed on clinical and imaging examinations. For patients who received Induction Chemotherapy (IC), the soft tissue area of the GTVnx was delineated after IC, and the skull base bones were delineated based on imaging examinations before IC. GTVnd refers to the gross tumor volume involving lymph nodes observed in clinical and imaging examinations. For patients who received IC, the GTVnd was delineated after IC. CTV1 refers to the clinical target volume 1, including GTVnx and the surrounding subclinical lesion area (expanded 5 mm around GTVnx), and also includes tumor shrinkage of the soft tissue area after IC. CTV2 refers to the clinical target volume 2, including CTV1 and its surrounding 5 mm dilatation, GTVnd, and the potential cervical lymph node metastasis area.

Dosages for primary and subclinical nasopharyngeal lesions were 68–76 Gy/30–33f for Planning Target Volume (PTV)-GTVnx, 60–64 Gy/30–33f for PTV-CTV1, and 50–54 Gy/30–33f for PTV-CTV2. The doses for cervical lymph nodes and cervical lymphatic drainage areas were 66–70 Gy/30–33f for PTV-GTVnd and 50–54 Gy/30–33f for PTV-CTV2. The limited dose refers to the Quantitative Analyses of Normal Tissue Effects in the Clinic (2012 standard).

#### 2.2.2. Systemic Treatment

Platinum-based Concurrent Chemoradiation Therapy (CCRT) is recommended for locally advanced nasopharyngeal carcinoma. Concurrent chemotherapy regimens included cisplatin (100 mg/m^2^) every three weeks or cisplatin (40 mg/m^2^) weekly during radiotherapy. 

IC regimens recommended by Chinese Society of Clinical Oncology guidelines [16] for nasopharyngeal carcinoma were as follows: TPF (docetaxel 60 mg/m^2^ d 1, cisplatin 60 mg/m^2^ d 1, and fluorouracil 600 mg/m^2^ per day, d 1–5, every 3 weeks) [17,18]; GP (gemcitabine 1000 mg/m^2^ d 1 and d 8 and cisplatin 80 mg/m^2^ d 1, every 3 weeks). 

Nimotuzumab, a humanized anti-EGFR monoclonal antibody, was approved by the Food and Drug Administration of China in combination with radiotherapy for stage III/IV NPC with positive EGFR expression. Patients were intravenously administered 200 mg of nimotuzumab [19,20] combined with concurrent chemoradiotherapy once weekly.

### 2.3. Extraction of Dosimetric Parameters

**Figure 1:**
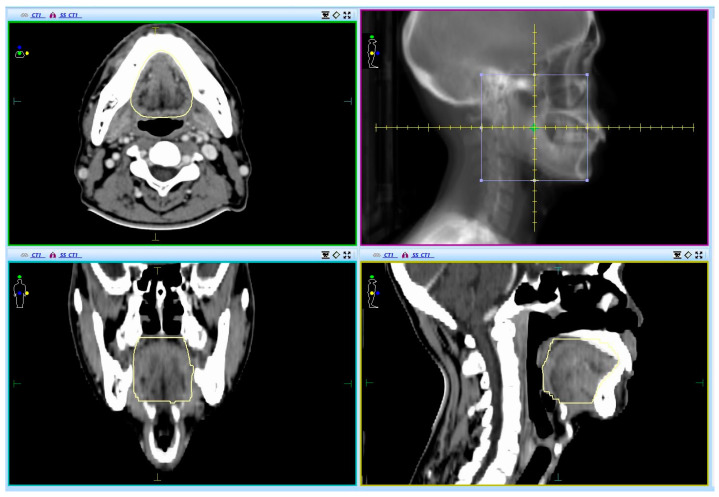
Delineation of the oral cavity in computed tomography scan.

Oral cavities in this study were contoured on the basis of contrast-enhanced CT images. As shown in Figure 1, the delineation of an oral cavity is as follows: the upper boundary is the hard palate, the anterior boundary reaches the buccal mucosa around the teeth, the lower boundary is the floor of the mouth, and the posterior boundary reaches the tongue surface and uvula [21]. No margins were added during oral cavity treatment. Dosimetric parameters were extracted from the Monaco 5.11 radiotherapy planning system (Electa, Stockholm, Sweden). The pretreatment parameters included the mean dose administered to the bilateral parotid glands and bilateral submandibular glands, V5, V10, V15, V20, V25, V30, V35, V40, V45, V50, V55, V60, V65, and V70, and the average and maximum doses of oral cavity.

### 2.4. OM Assessment 

Mucositis toxicity in patients was assessed according to the WHO mucosal acute response criteria: grade 0, no mucositis; grade 1, sore throat, but no ulcers on oral mucosa and tongue, and able to eat solid food; grade 2, ulcers on oral mucosa or tongue, able to eat solid food; grade 3, ulcers with extensive erythema and liquid diet only; and grade 4, ulceration, unable to eat through the mouth, requires feeding through nasogastric tube or gastrostomy enteral nutrition or parenteral feeding [22]. Physicians and nurses who received unified training were appointed as observers to record the OM of patients at least once a week during IMRT. According to the highest grade of OM during radiotherapy, the enrolled patients were divided into two groups: with SOM (score ≥ 3) and without SOM (score < 3).

### 2.5. Statistical Analysis

The clinical characteristics and dosimetric parameters of the two groups were compared. Student’s *t*-test was used to compare the continuous characteristics of age, Body Mass Index (BMI) and dosimetry parameters, and Mann–Whitney U test was used to compare variables with non-normal distributions.

The chi-squared test or Fisher’s exact test was used to compare categorical characteristics of gender, smoking, T stage, N stage, IC, nimotuzumab, immunotherapy, diabetes, and cumulative cisplatin dose during Concurrent Chemoradiotherapy (CCD). 

Least Absolute Shrinkage and Selection Operator (LASSO) penalized regression analysis was used to screen important variables with non-zero coefficients, which could then be used as potential predictors of SOM prediction models. Based on the above results of LASSO regression analysis, multiple logistic regression analysis was used to further identify the independent predictors of SOM, and a nomogram was developed. The calibration was evaluated by constructing a calibration curve. The Area Under the Curve (AUC) was used to assess the discriminating ability of the nomogram.

Statistical analyses were performed using SPSS Statistics Version 25.0 software (IBM Co., Armonk, NY, USA) and R version 3.5.3 (http://www.r-project.org/, accessed on 25 November 2020). All *p* values were two-sided. Statistical significance was set at *p* < 0.05.

## 3. Results

### 3.1. Patient Characteristics

**Figure 2:**
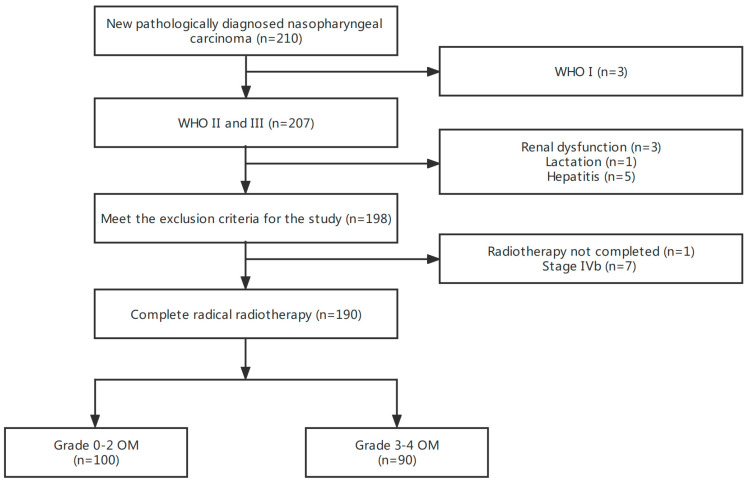
Flowchart depicting patient selection.

In this study, we planned to screen 210 NPC patients and finally enrolled 190 patients: 100 patients had OM of grade 0–2 during radiotherapy (without SOM), and the other 90 patients had OM of grade 3–4 (with SOM). The overall incidence rate of SOM was 47.4%. The patient screening process is illustrated in Figure 2. The clinical characteristics of these two groups of patients are shown in Table 1. Except for the T stage, the baseline clinical characteristics of these two groups of patients were well balanced. There appeared to be more patients with SOM undergoing IC than those without SOM (71.1% vs. 59.0%), but the difference was not statistically significant.

Student’s *t*-test was used to compare the continuous characteristics in age and Body Mass Index (BMI). The chi-squared test or Fisher’s exact test was used to compare categorical characteristics of gender, smoking, T stage, N stage, IC, nimotuzumab, immunotherapy, diabetes, and cumulative cisplatin dose during Concurrent Chemoradiotherapy (CCD).

### 3.2. Dosimetry Parameters of the Oral Mucosa and Salivary Glands

The dosimetry parameters of radiotherapy were comparable between the patients with and without SOM (Table 2). The Dmean of the right parotid, Dmean and V20–V65 of the oral cavity in patients with SOM were significantly higher than those in patients without SOM (all of the above indicators, *p* < 0.05). No significant differences were found in V5–V15, V70, and Dmax of the oral cavity between patients with and without SOM. The prediction probabilities of the dosimetric parameters are listed in Table 2. From Table 2, we can conclude that the assessment ability of every single parameter is low (less than 0.700).

### 3.3. Identification of Independent Predictors of SOM

**Figure 3:**
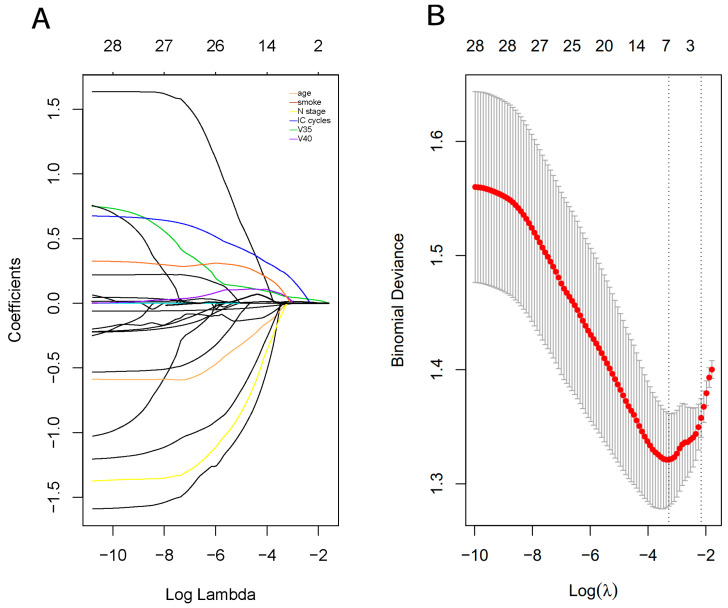
Parameters selection using LASSO logistic regression model. (**A**) LASSO coefficient profiles of parameters for SOM. A coefficient profile plot is produced against the log(λ) sequence. A vertical line is drawn at the value selected using 10-fold cross-validation, where optimal λ results in 3 nonzero coefficients. (**B**) Tuning parameter (λ) selection in the LASSO model uses 10-fold cross-validation via minimum criteria. Binomial deviances from the LASSO regression cross-validation procedure are plotted as a function of log (λ). Dotted vertical lines are drawn at the optimal values by using the minimum criteria and the 1-minus-standard-error of the minimum criteria (the 1-SE criteria).

The results of the univariate analysis indicate that T stage, N stage, IC cycles, V25–V60, Dmean, and Dmax of the oral cavity were significantly different between these two groups with and without SOM. LASSO logistic regression analysis was then performed based on the aforementioned factors, with obvious differences (*p <* 0.1). Using the LASSO method, age, N stage, IC cycles, and V40 and V35 of the oral cavity were screened (Figure 3A,B). Thereafter, a multivariate logistic regression analysis based on these parameters was performed. The results show that age (*p* = 0.077), N stage (*p* = 0.018), IC cycles (*p* = 0.035), and V40 (*p* = 0.038) of the oral cavity were independent predictors of SOM after IMRT. 

### 3.4. Construction and Validation of A Nomogram for Predicting SOM

**Figure 4:**
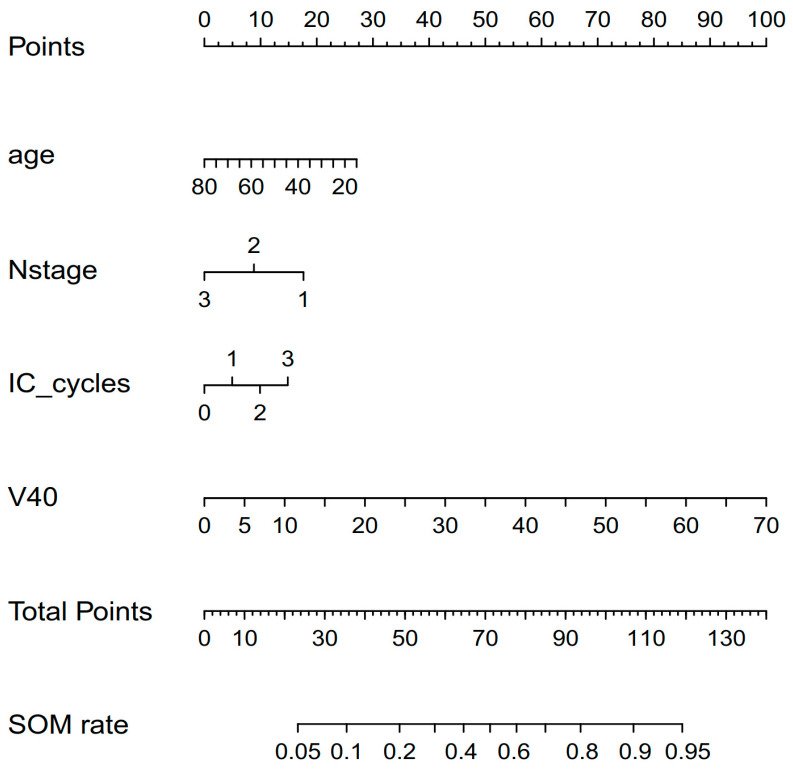
Nomogram for the risk incidence of SOM in patients with NPC during IMRT. Variables include age, N stage, IC cycles, and V40 of the oral cavity.

**Figure 5:**
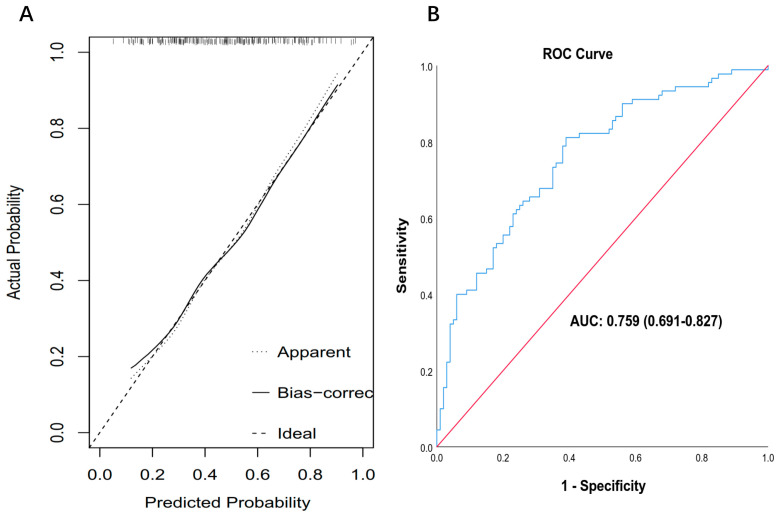
The calibration curves and receiver operating curves of the nomogram. (**A**) The calibration curves of the nomogram. The Y-axis represents the actual SOM rate. The X-axis represents the predicted SOM risk. The dotted line represents a perfect prediction, and the solid line represents the predictive performance of the nomogram. The closer the solid line fits to the dotted line, the better the predictive accuracy of the nomogram is. (**B**) The receiver operating curves of the nomogram. The area under the receiver operating characteristic curve was 0.759.

Based on the variables screened using multivariate logistic regression analysis (including age, N stage, IC cycles, and V40 of the oral cavity), a nomogram capable of predicting the probability of SOM was constructed. By summing the scores for each factor of individual patients and locating the total score on the scoring scale, the probability of SOM for each NPC patient undergoing IMRT could be predicted (Figure 4). The calibration curve of the nomogram is shown in Figure 5A. The calibration curve showed good calibration. The AUC value of 0.759 (95% Confidence Interval: 0.691–0.827) shown by the nomogram also indicated good discrimination (Figure 5B).

## 4. Discussion

OM is one of the common complications with a significant impact on quality of life in patients with head and neck tumors, especially in those with NPC, during the course of radiotherapy. Currently, IMRT has replaced conventional radiotherapy as the mainstream therapy for NPC because of its better dose distribution and better protection of surrounding tissues [12]. However, numerous studies have shown that the incidence of OM remains high in patients with NPC treated with IMRT, with approximately 50% of patients developing SOM [13]. One possible reason is that there is no rigid limitation of the oral cavity dosage in IMRT. Another possible reason is that chemotherapy (including IC and concurrent chemotherapy), EGFR monoclonal antibody, and immunotherapy, may affect the occurrence of OM under current treatment modalities. From a clinical point of view, SOM, defined as grade 3–4 OM according to the WHO scale, is particularly important. The consequences of SOM include development of eating disorders, the need for intravenous nutrition, administration of antibiotics, or use of opioid analgesics, which are associated with longer hospital stays and higher costs [23]. In addition, radiotherapy was interrupted due to SOM in about 11% of patients [8]. Previous studies have shown that unplanned interruption of radiotherapy for one day can reduce the tumor control rate by at least 1% [24]. Based on the above reasons, we believe that the early identification of high-risk individuals who may develop SOM is critical for disease prevention and intervention, thus possibly reducing the incidence of SOM in NPC. This study found that age, N stage, IC cycles, and V40 of the oral cavity were independent predictors of SOM, whereas other clinical factors, including nimotuzumab, concurrent chemotherapy, T stage, and immunotherapy, had no effect on the occurrence of SOM. Based on these independent predictors, a nomogram was established to predict the risk of SOM occurrence.

Although many assessment scales based on morphological or functional changes are currently employed to grade OM severity, there is no standard scale for assessing OM recognized by all researchers. An ideal assessment tool should have the following characteristics: it is convenient for clinical practice, it has clear differences between different levels, and it can accurately reflect the adverse side effects on patients. The most commonly utilized scales include CTC version 4.0, RTOG, EORTC, and the criteria set out by the WHO and OMAS [8,9]. According to the criteria of an ideal OM evaluation tool, we adopted the WHO scale in this study since the WHO oral toxicity scale can combine changes in mucosal morphology and dysfunction.

The widespread use of concurrent chemotherapy in head and neck tumors over the past few decades has improved the efficacy of radiotherapy but also caused higher rates of toxicity [17,25,26]. According to several investigators, the frequency, severity, and duration of grade 3–4 OM in patients with head and neck tumors who received concurrent chemoradiation were higher than those in patients who received radiotherapy alone. The incidence of grade 3 and grade 4 OM in patients with Head and Neck Cancer Patients (HNCPs) receiving concurrent chemoradiotherapy was reported to be 80% and 39%, respectively [27,28]. Elting et al. [13] reported a similar result, with an overall SOM incidence of 66% in HNCPs treated with chemoradiotherapy. This study showed that 47.8% of patients with NPC who received concurrent chemoradiotherapy developed SOM, which was similar to the results of the above studies. However, further statistical analysis showed that concurrent chemotherapy was not an independent predictor of SOM in NPC patients treated with IMRT, regardless of the cumulative cisplatin dose. A possible explanation is that this is related to the low proportion of patients who received radiotherapy alone in this study.

According to the National Comprehensive Cancer Network guidelines for NPC, the currently recommended treatment for stage III–IVa NPC is IC followed by concurrent chemoradiotherapy. However, whether cycles of IC affect the incidence and severity of OM during subsequent radiotherapy remains inconclusive. Zhang et al. [26] found that the incidence of severe mucositis between the GP regimen IC group and the standard therapy group did not reach a statistically significant difference (28.9% vs. 32.1%). Similar to their study, in another randomized controlled trial of IC for NPC conducted by Sun et al. [17], the incidences of SOM in the TPF regimen plus concurrent chemoradiotherapy groups and concurrent chemoradiotherapy alone groups were 41% and 35%, respectively. Although there was a trend toward an increased incidence of SOM in the IC group, the difference between these two groups was not statistically significant (*p* = 0.2). Hu et al. [29] conducted a meta-analysis to analyze the benefit of IC for stage III–IV NPC, and the results suggest that the incidence of grade 2 or above OM in the concurrent chemoradiotherapy group was significantly lower than that in the IC plus CCRT group (Odds Ratio (OR), 1.05 (95% CI, 0.71–1.56), *p* = 0.023). This study found that IC increased the incidence of OM. In particular, an increased number of IC cycles is associated with the degree of OM elevation. Multivariate logistic regression analysis confirmed that the number of chemotherapy cycles was an independent predictor of OM (OR, 1.338; 95% CI, 1.021–1.1.754; *p* = 0.035).

In addition to standard radiotherapy and chemotherapy, more targeted drugs, such as nimotuzumab and cetuximab, are increasingly being used in clinical practice for the management of NPC. The target specificity of these new drugs makes them more effective and less toxic than standard chemotherapy. However, clinicians are faced with new toxicities because the mechanisms of targeted drugs and chemotherapeutic drugs are very different. Currently, the effect of EGFR-binding monoclonal antibodies on oral mucositis during radiotherapy remains controversial. Chen et al. [30] showed that the incidence of oral mucositis was significantly higher in patients treated with nimotuzumab during radiotherapy than that in those who were not. Soutome et al. [31] reported similar findings in oral cancer. However, other studies have yielded conflicting results. In randomized trials conducted by Bonner et al. [32], cetuximab was added to radiotherapy to significantly improve prognosis such as locoregional progression, progression-free survival, and overall survival without aggravating common adverse events, including OM. Specifically, 52% and 56% of patients with HNCP developed grade 3–4 OM in the RT alone and RT plus cetuximab groups. Magrini et al. [33] conducted a phase II trial to study the efficacy and adverse effects of concomitant cetuximab versus cisplatin in locally advanced head and neck cancer. Although the incidence of severe cutaneous toxicity of G3 or worse was significantly higher in the cetuximab arm than that in the cisplatin arm, there was no difference in the incidence of acute mucositis between the two groups. Of the 190 patients with NPC enrolled in this study, 46 (24.2%) were treated with nimotuzumab. This study found that SOM occurred in 27.0% and 21.1% (*p* = 0.344) of NPC patients in the radiotherapy alone group and in the radiotherapy plus nimotuzumab group, respectively, suggesting that nimotuzumab did not increase the incidence of SOM in NPC patients undergoing IMRT. This contributes to resolving the current controversies mentioned above. On the other hand, it suggests that for patients with locally advanced NPC, it may not be appropriate not to use nimotuzumab due to concerns about possible acute radiation OM.

It is well known that inadequate nutritional intake during radiotherapy in HNCPs impairs the multifaceted process of mucosal ulcer healing, thereby impairing ulcerative mucosal healing. Several studies have found that a low BMI is associated with poor survival in HNCPs [34,35]. In addition, a lower BMI means a higher incidence of grade 2–3 mucositis in HNCPs treated with RT [36]. Therefore, this study included pre-treatment BMI in the analysis. We showed that patients with SOM had a lower BMI than those without SOM, but the difference was not significant. Age and sex are risk factors that may affect the occurrence of SOM. Several studies have suggested that females are more likely to develop mucositis grades 3–4 than males [37]. Similar results were observed in this study, but they were not statistically significant. There is no consensus regarding this issue. There is no consensus on whether age is an independent risk factor for SOM. Pico et al. [38] reported that older patients with head and neck tumors tended to have a higher risk of developing mucositis than did younger patients during radiotherapy. Our findings are consistent with these observations. 

To date, no randomized clinical trial has investigated the effects of immunotherapy on oral radiation mucositis. In recurrent or metastatic nasopharyngeal carcinoma, GP combined immunotherapy is one of the recommended treatments [39,40]. We cautiously performed immunotherapy combined with induction chemotherapy in a small number of patients with nasopharyngeal carcinoma in T4NxM0 or TxN3M0 stages with the approval of the Ethics Committee. For these patients, we are very concerned about whether the addition of immunotherapy would aggravate the side effects of radiotherapy. Based on the above considerations, we included these patients in this study for analysis. This retrospective study did not find toxic effect of immunotherapy on SOM. In view of the fact that only 31 (16.3%) patients in this study received immunotherapy, future research is needed on this topic.

Common non-drug preventive measures, such as quitting smoking, quitting drinking, avoiding irritating food, and maintaining oral hygiene, should be taken regardless of patients’ risk of developing SOM in clinical practice for patients receiving radiotherapy to head and neck [41,42]. For patients with high risk of SOM, more radical measures should be taken. When designing the radiotherapy plan, minimize the exposure volume and dose of oral cavity. Tongue displacement device and individual three-dimensional printed mold can be used to assist in decreasing the radiation dose to tongue [43,44]. Poor nutritional status is one of the main factors for poor prognosis of nasopharyngeal carcinoma [45]. So, early nutritional support treatment, especially the establishment of enteral nutrition pathway, is very important to improve the prognosis of patients at high risk of SOM. In addition, benzydamine [46] and physiological saline gargling [47] are also recommended options in preventing SOM based on evidence from clinical studies. In a clinical trial initiated by us on thalidomide in the prevention and treatment of oral mucositis in patients with NPC during CCRT, thalidomide showed good efficacy and safety [48]. Considering that the progression and aggravation of radiation oral mucositis is associated with shifts in the oral microbiota, interventions aimed at changes in oral microbial populations are also research directions worthy of attention [49,50].

In this study, we used age, N stage, IC cycles, and V40 of the oral cavity to establish a nomogram to predict the probability of SOM during IMRT in patients with NPC. Moreover, the nomogram has relatively good predictive ability. However, this study has some limitations. First, the scope of application of this nomogram is limited because this study was based on data from patients with NPC undergoing IMRT. The predictive power of the model could not be determined for patients with head and neck tumors other than NPC. Given that immunotherapy is still at the stage of clinical exploratory research in the treatment of NPC, this study did not stratify the specific types of immunotherapy, which may have an impact on the results. Additionally, the lack of external verification is an unavoidable limitation of this study. Further studies with a larger sample size on our prediction signatures and their associations with SOM are needed in the future. 

## 5. Conclusions

A predictive nomogram for SOM in patients with NPC during radiotherapy was established and validated in this study. The nomogram provides a somewhat reliable and practical model for clinically predicting the probability of SOM in patients with NPC before IMRT. This enables physicians to identify patients at high risk of SOM to enable earlier intervention, thereby improving radiotherapy compliance and quality of life during radiotherapy among patients with NPC. 

## Figures and Tables

**Table 1:** Characteristics between patients with and without SOM.

Characteristics	Without SOM	With SOM	*p*
	No (%)	No (%)	
Age, years #	47.43 ± 11.202	45.11 ± 11.769	0.166
Gender			0.113
Male	76 (76.0%)	59 (65.6%)	
Female	24 (24.0%)	31 (34.4%)	
Smoking			0.073
Yes	53 (53.0%)	36 (40.0%)	
No	47 (47.0%)	54 (60.0%)	
T classification			0.015
T1	3 (3.0%)	2 (2.2%)	
T2	18 (18.0%)	17 (18.9%)	
T3	49 (49.0%)	26 (28.9%)	
T4	30 (30.0%)	45 (50.0%)	
N classification			0.179
N0	4 (4.0%)	6 (6.7%)	
N1	34 (34.0%)	41 (45.6%)	
N2	32 (32.0%)	18 (20.0%)	
N3	30 (30.0%)	25 (27.8%)	
IC cycles			0.137
0	41 (41.0%)	26 (28.9%)	
1	8 (8.0%)	4 (4.4%)	
2	31 (31.0%)	32 (35.6%)	
3	20 (20.0%)	28 (31.1%)	
CCRT			0.281
No	8 (8.0%)	6 (6.7%)	
CCD ≥ 200 mg/m^2^	44 (44.0%)	50 (55.6%)	
CCD < 200 mg/m^2^	48 (48.0%)	34 (37.8%)	
Nimotozumab			0.344
Yes	27 (27.0%)	19 (21.1%)	
No	73 (73.0%)	71 (78.9%)	
Immunotherapy			0.605
Yes	15 (15.0%)	16 (17.8%)	
No	85 (85.0%)	74 (82.2%)	
BMI	25.15 ± 3.43	22.29 ± 3.27	0.781
Diabetes			0.724
Yes	5 (5.0%)	3 (3.3%)	
No	95 (95.0%)	87 (96.7%)	

IC: Induction Chemotherapy. CCRT: Concurrent Chemoradiothrapy. BMI: Body Mass Index. CCD: Cumulative Cisplatin Dose. # Mean ± standard deviation.

**Table 2:** Dose-volume parameters between patients with and without SOM.

Parameters	Dosimetry Parameters (Median, Range)			Predicted Probability		
	Without SOM	With SOM	*p*	AUC	95%CI	*p*
Oral mucosa Vx (%)						
V5	100 (100–100)	100 (100–100)	1.000	0.500	0.418–0.582	1.000
V10	100 (100–100)	100 (100–100)	1.000	0.500	0418–0.582	1.000
V15	100 (98.8–100)	100 (98.0–100)	0.306	0.526	0.444–0.608	0.541
V20	97.6 (92.3–100)	98.7 (92.0–100)	0.021	0.597	0.515–0.678	0.022
V25	89.05 (72.9–99.4)	91.85 (75.2–100)	0.003	0.626	0.545–0.706	0.003
V30	73.25 (46.6–90.6)	77.7 (47.9–94)	<0.0001	0.649	0.571–0.728	<0.0001
V35	42.35 (18.6–68.1)	49.35 (23.5–84.1)	<0.0001	0.677	0.601–0.753	<0.0001
V40	22.3 (4.3–53)	29.4 (9.4–65.4)	<0.0001	0.697	0.623–0.772	<0.0001
V45	12.65 (1.0–38.6)	17.45 (3.6–50.4)	<0.0001	0.695	0.621–0.770	<0.0001
V50	6.9 (0.1–28.2)	10.8 (0.6–39.4)	<0.0001	0.690	0.615–0.765	<0.0001
V55	3.4 (0–20.4)	6.05 (0–30.3)	<0.0001	0.672	0.595–0.748	<0.0001
V60	1.2 (0–14.6)	2.7 (0–21.9)	0.001	0.640	0.561–0.719	0.001
V65	0.2 (0–9.9)	0.75 (0–14.5)	0.008	0.609	0.528–0.689	0.010
V70	0 (0–5.1)	0 (0–7.5)	0.052	0.571	0.489–0.652	0.094
Oral mucosa Dx (Gy)						
Dmean	35.02 (28.60–42.70)	36.93 (30.82–47.54)	<0.0001	0.696	0.621–0.770	<0.0001
Dmax	68.05 (55.23–76.35)	69.77 (57.72–77.85)	0.053	0.581	0.500–0.662	0.053
Dmean for other structures						
Parotid_L	35.19 (25.54–71.69)	35.33 (25.73–62.64)	0.169	0.558	0.476–0.640	0.169
Parotid_R	34.98 (26.85–68.33)	36.13 (27.51–64.91)	0.025	0.595	0.513–0.676	0.025
Submandibular_L	56.85 (45.28–71.57)	56.86 (44.38–70.89)	0.926	0.504	0.421–0.586	0.926
Submandibular_R	57.20 (45.59–71.85)	57.54 (45.89–72.18)	0.632	0.520	0.438–0.602	0.632

Student’s *t*-test was used to compare the continuous characteristics in dosimetry parameters, and Mann–Whitney U test was used to compare variables with abnormal distributions. After Bonferroni correction, only *p* lower than 2.50 × 10^−3^ (0.05/20 parameters) were considered statistically significant. The Area Under the Curve (AUC) of the Receiver Operating Characteristic (ROC) curve was used to assess the predicted probability.

## Data Availability

The original contributions presented in the study are included in the article. Further inquiries can be directed to the corresponding author.

## References

[1] Bray F., Ferlay J., Soerjomataram I., Siegel R.L., Torre L.A., Jemal A. (2018). Global cancer statistics 2018: GLOBOCAN estimates of incidence and mortality worldwide for 36 cancers in 185 countries. CA Cancer J. Clin..

[2] Cao W., Chen H.-D., Yu Y.-W., Li N., Chen W.-Q. (2021). Changing profiles of cancer burden worldwide and in China: A secondary analysis of the global cancer statistics 2020. Chin. Med. J..

[3] Wu F., Wang R., Lu H., Wei B., Feng G., Li G., Liu M., Yan H., Zhu J., Zhang Y. (2014). Concurrent chemoradiotherapy in locoregionally advanced nasopharyngeal carcinoma: Treatment outcomes of a prospective, multicentric clinical study. Radiother. Oncol..

[4] Luo D.H., Hong M.H., Guo L., Cao K.J., Deng M.Q., Mo H.Y. (2005). Analysis of oral mucositis risk factors during radiotherapy for nasopharyngeal carcinoma patients and establishment of a discriminant model. Ai Zheng = Aizheng = Chin. J. Cancer.

[5] Wu S., Cui T., Zhao C., Pan J., Xu B., Tian Y., Cui N. (2008). A randomized controlled multicenter trial of Actovegin against acute oral mucositis induced by chemo-radiotherapy for nasopharyngeal carcinoma. Int. J. Radiat. Oncol. Biol. Phys..

[6] Tao Z., Gao J., Qian L., Huang Y., Zhou Y., Yang L., He J., Yang J., Wang R., Zhang Y. (2017). Factors associated with acute oral mucosal reaction induced by radiotherapy in head and neck squamous cell carcinoma: A retrospective single-center experience. Medicine.

[7] Zhu X.-X., Yang X.-J., Chao Y.-L., Zheng H.-M., Sheng H.-F., Liu H.-Y., He Y., Zhou H.-W. (2017). The Potential Effect of Oral Microbiota in the Prediction of Mucositis During Radiotherapy for Nasopharyngeal Carcinoma. eBioMedicine.

[8] Sonis S.T., Elting L.S., Keefe D., Peterson D.E., Schubert M., Hauer-Jensen M., Bekele B.N., Raber-Durlacher J., Donnelly J.P., Rubenstein E.B. (2004). Perspectives on cancer therapy-induced mucosal injury: Pathogenesis, measurement, epidemiology, and consequences for patients. Cancer.

[9] Rosenthal D.I., Mendoza T.R., Chambers M.S., Asper J.A., Gning I., Kies M.S., Weber R.S., Lewin J., Garden A., Ang K.K. (2007). Measuring head and neck cancer symptom burden: The development and validation of the M. D. Anderson Symptom Inventory, head and neck module. Head Neck.

[10] Anderson C.M., Lee C., Saunders D., Curtis A., Dunlap N.E., Nangia C., Lee A., Holmlund J., Brill J., Sonis S.T. (2018). A Randomized, Placebo (PBO) Controlled, Double-Blind P2b Trial of GC4419 (Avisopasem Manganese) to Reduce Severe Radiation-Related Oral Mucositis (SOM) in Patients (pts) with Locally Advanced Squamous Cell Cancer of the Oral Cavity (OC) or Oropharynx (OP). Int. J. Radiat. Oncol. Biol. Phys..

[11] Hua X., Chen L.-M., Zhu Q., Hu W., Lin C., Long Z.-Q., Wen W., Sun X.-Q., Lu Z.-J., Chen Q.-Y. (2019). Efficacy of controlled-release oxycodone for reducing pain due to oral mucositis in nasopharyngeal carcinoma patients treated with concurrent chemoradiotherapy: A prospective clinical trial. Support. Care Cancer.

[12] Au K.H., Ngan R.K.C., Ng A.W.Y., Poon D.M.C., Ng W.T., Yuen K.T., Lee V.H.F., Tung S.Y., Chan A.T.C., Sze H.C.K. (2018). Treatment outcomes of nasopharyngeal carcinoma in modern era after intensity modulated radiotherapy (IMRT) in Hong Kong: A report of 3328 patients (HKNPCSG 1301 study). Oral. Oncol..

[13] Elting L.S., Cooksley C.D., Chambers M.S., Garden A.S. (2007). Risk, outcomes, and costs of radiation-induced oral mucositis among patients with head-and-neck malignancies. Int. J. Radiat. Oncol. Biol. Phys..

[14] Li K., Yang L., Hu Q.-Y., Chen X.-Z., Chen M., Chen Y. (2017). Oral Mucosa Dose Parameters Predicting Grade >= 3 Acute Toxicity in Locally Advanced Nasopharyngeal Carcinoma Patients Treated With Concurrent Intensity-Modulated Radiation Therapy and Chemotherapy: An Independent Validation Study Comparing Oral Cavity versus Mucosal Surface Contouring Techniques. Transl Oncol..

[15] Wang R., Kang M. (2021). Guidelines for radiotherapy of nasopharyngeal carcinoma. Precis. Radiat. Oncol..

[16] Tang L.-L., Chen Y.-P., Chen C.-B., Chen M.-Y., Chen N.-Y., Chen X.-Z., Du X.-J., Fang W.-F., Feng M., Gao J. (2021). The Chinese Society of Clinical Oncology (CSCO) clinical guidelines for the diagnosis and treatment of nasopharyngeal carcinoma. Cancer Commun..

[17] Sun Y., Li W.-F., Chen N.-Y., Zhang N., Hu G.-Q., Xie F.-Y., Sun Y., Chen X.-Z., Li J.-G., Zhu X.-D. (2016). Induction chemotherapy plus concurrent chemoradiotherapy versus concurrent chemoradiotherapy alone in locoregionally advanced nasopharyngeal carcinoma: A phase 3, multicentre, randomised controlled trial. Lancet Oncol..

[18] Li W.-F., Chen N.-Y., Zhang N., Hu G.-Q., Xie F.-Y., Sun Y., Chen X.-Z., Li J.-G., Zhu X.-D., Hu C.-S. (2019). Concurrent chemoradiotherapy with/without induction chemotherapy in locoregionally advanced nasopharyngeal carcinoma: Long-term results of phase 3 randomized controlled trial. Int. J. Cancer.

[19] You R., Sun R., Hua Y.-J., Li C.-F., Li J.-B., Zou X., Yang Q., Liu Y.-P., Zhang Y.-N., Yu T. (2017). Cetuximab or nimotuzumab plus intensity-modulated radiotherapy versus cisplatin plus intensity-modulated radiotherapy for stage II-IVb nasopharyngeal carcinoma. Int. J. Cancer.

[20] You R., Hua Y.-J., Liu Y.-P., Yang Q., Zhang Y.-N., Li J.-B., Li C.-F., Zou X., Yu T., Cao J.-Y. (2017). Concurrent Chemoradiotherapy with or without Anti-EGFR-Targeted Treatment for Stage II-IVb Nasopharyngeal Carcinoma: Retrospective Analysis with a Large Cohort and Long Follow-up. Theranostics.

[21] Brouwer C.L., Steenbakkers R.J.H.M., Bourhis J., Budach W., Grau C., Gregoire V., van Herk M., Lee A., Maingon P., Nutting C. (2015). CT-based delineation of organs at risk in the head and neck region: DAHANCA, EORTC, GORTEC, HKNPCSG, NCIC CTG, NCRI, NRG Oncology and TROG consensus guidelines. Radiother. Oncol..

[22] Sonis S.T. (2013). Oral mucositis in head and neck cancer: Risk, biology, and management. Am. Soc. Clin. Oncol. Educ. Book.

[23] Pulito C., Mori F., Sacconi A., Casadei L., Ferraiuolo M., Valerio M.C., Santoro R., Goeman F., Maidecchi A., Mattoli L. (2015). Cynara scolymus affects malignant pleural mesothelioma by promoting apoptosis and restraining invasion. Oncotarget.

[24] Withers H.R., Taylor J.M.G., Maciejewski B. (1988). The hazard of accelerated tumor clonogen repopulation during radiotherapy. Acta Oncol..

[25] Chen L., Hu C.-S., Chen X.-Z., Hu G.-Q., Cheng Z.-B., Sun Y., Li W.-X., Chen Y.-Y., Xie F.-Y., Liang S.-B. (2012). Concurrent chemoradiotherapy plus adjuvant chemotherapy versus concurrent chemoradiotherapy alone in patients with locoregionally advanced nasopharyngeal carcinoma: A phase 3 multicentre randomised controlled trial. Lancet Oncol..

[26] Zhang Y., Chen L., Hu G.-Q., Zhang N., Zhu X.-D., Yang K.-Y., Jin F., Shi M., Chen Y.P., Hu W.-H. (2019). Gemcitabine and Cisplatin Induction Chemotherapy in Nasopharyngeal Carcinoma. N. Engl. J. Med..

[27] Haddad R., O’Neill A., Rabinowits G., Tishler R., Khuri F.R., Adkins D., Clark J., Sarlis N., Lorch J., Beitler J.J. (2013). Induction chemotherapy followed by concurrent chemoradiotherapy (sequential chemoradiotherapy) versus concurrent chemoradiotherapy alone in locally advanced head and neck cancer (PARADIGM): A randomised phase 3 trial. Lancet Oncol..

[28] Bossi P., Locati L.D., Licitra L. (2012). Palifermin in prevention of head and neck cancer radiation-induced mucositis: Not yet a definitive word on safety and efficacy profile. J. Clin. Oncol..

[29] Hu T., Fang L., Shi L., Wang W., Huang Y. (2021). Survival benefit of induction chemotherapy in treatment for stage III or IV locally advanced nasopharyngeal carcinoma—An updated meta-analysis and systematic review. Am. J. Otolaryngol..

[30] Chen X., Yao L., Shan Q., Qian X., Lu X., Tang X., Chen S., Yu W. (2021). Risk factors for oral mucositis in patients with malignant tumors: A prospective cohort study. Ann. Palliat. Med..

[31] Soutome S., Yanamoto S., Nishii M., Kojima Y., Hasegawa T., Funahara M., Akashi M., Saito T., Umeda M. (2021). Risk factors for severe radiation-induced oral mucositis in patients with oral cancer. J. Dent. Sci..

[32] Bonner J.A., Harari P.M., Giralt J., Azarnia N., Shin D.M., Cohen R.B., Jones C.U., Sur R., Raben D., Jassem J. (2006). Radiotherapy plus cetuximab for squamous-cell carcinoma of the head and neck. N. Engl. J. Med..

[33] Magrini S.M., Buglione M., Corvo R., Pirtoli L., Paiar F., Ponticelli P., Petrucci A., Bacigalupo A., Crociani M., Lastrucci L. (2016). Cetuximab and Radiotherapy Versus Cisplatin and Radiotherapy for Locally Advanced Head and Neck Cancer: A Randomized Phase II Trial. J. Clin. Oncol..

[34] Ottosson S., Söderström K., Kjellén E., Nilsson P., Zackrisson B., Laurell G. (2014). Weight and body mass index in relation to irradiated volume and to overall survival in patients with oropharyngeal cancer: A retrospective cohort study. Radiat. Oncol..

[35] Pai P.-C., Chuang C.-C., Tseng C.-K., Tsang N.-M., Chang K.-P., Yen T.-C., Liao C.-T., Hong J.-H., Chang J.T.-C. (2012). Impact of pretreatment body mass index on patients with head-and-neck cancer treated with radiation. Int. J. Radiat. Oncol. Biol. Phys..

[36] Arrieta-Blanco J.J., Bartolomé-Villar B., Jiménez-Martinez E., Saavedra-Vallejo P., Arrieta-Blanco F.J. (2003). Dental problems in patients with diabetes mellitus (II): Gingival index and periodontal disease. Med. Oral..

[37] Vokurka S., Bystricka E., Koza V., Scudlová J., Pavlicová V., Valentová D., Visokaiová M., Misaniová L. (2006). Higher incidence of chemotherapy induced oral mucositis in females: A supplement of multivariate analysis to a randomized multicentre study. Support. Care Cancer.

[38] Pico J.-L., Avila-Garavito A., Naccache P. (1998). Mucositis: Its Occurrence, Consequences, and Treatment in the Oncology Setting. Oncologist.

[39] Mai H.-Q., Chen Q.-Y., Chen D., Hu C., Yang K., Wen J., Li J., Shi Y.-R., Jin F., Xu R. (2021). Toripalimab or placebo plus chemotherapy as first-line treatment in advanced nasopharyngeal carcinoma: A multicenter randomized phase 3 trial. Nat. Med..

[40] Yang Y., Qu S., Li J., Hu C., Xu M., Li W., Zhou T., Shen L., Wu H., Lang J. (2021). Camrelizumab versus placebo in combination with gemcitabine and cisplatin as first-line treatment for recurrent or metastatic nasopharyngeal carcinoma (CAPTAIN-1st): A multicentre, randomised, double-blind, phase 3 trial. Lancet Oncol..

[41] Bjarnason G.A., MacKenzie R.G., Nabid A., Hodson I.D., El-Sayed S., Grimard L., Brundage M., Wright J., Hay J., Ganguly P. (2009). Comparison of Toxicity Associated with Early Morning Versus Late Afternoon Radiotherapy in Patients with Head-and-Neck Cancer: A Prospective Randomized Trial of the National Cancer Institute of Canada Clinical Trials Group (Hn3). Int. J. Radiat. Oncol. Biol. Phys..

[42] (2008). NCCN Task Force Report: Prevention and Management of Mucositis in Cancer Care. J. Natl. Compr. Canc. Netw..

[43] Lancellotta V., Pagano S., Tagliaferri L., Piergentini M., Ricci A., Montecchiani S., Saldi S., Chierchini S., Cianetti S., Valentini V. (2019). Individual 3-dimensional printed mold for treating hard palate carcinoma with brachytherapy: A clinical report. J. Prosthet. Dent..

[44] Hong C.-S., Oh D., Ju S.G., Ahn Y.C., Kim Y.-B., Park S., Lee W. (2021). Tongue Displacement Device in Decreasing the Radiation Dose to Tongue and Preventing Proton Beam Overshoot in Proton Therapy for Unilateral Head and Neck Cancer. Front. Phys.

[45] Rabinovitch R., Grant B., Berkey B.A., Raben D., Ang K.K., Fu K.K., Cooper J.S. (2006). Impact of nutrition support on treatment outcome in patients with locally advanced head and neck squamous cell cancer treated with definitive radiotherapy: A secondary analysis of RTOG trial 90-03. Head Neck.

[46] Kazemian A., Kamian S., Aghili M., Hashemi F.A., Haddad P. (2009). Benzydamine for prophylaxis of radiation-induced oral mucositis in head and neck cancers: A double-blind placebo-controlled randomized clinical trial. Eur. J. Cancer Care.

[47] Peterson D.E. (2011). Management of oral and gastrointestinal mucositis: ESMO Clinical Practice Guidelines. Ann. Oncol..

[48] Liang L., Liu Z., Zhu H., Wang H., Wei Y., Ning X., Shi Z., Jiang L., Lin Z., Yan H. (2022). Efficacy and safety of thalidomide in preventing oral mucositis in patients with nasopharyngeal carcinoma undergoing concurrent chemoradiotherapy: A multicenter, open-label, randomized controlled trial. Cancer.

[49] Carli E., Pasini M., Lardani L., Giuca G., Miceli M. (2021). Impact of self-ligating orthodontic brackets on dental biofilm and periodontal pathogens in adolescents. J. Biol. Regul. Homeost. Agents.

[50] Hou J., Zheng H., Li P., Liu H., Zhou H., Yang X. (2018). Distinct shifts in the oral microbiota are associated with the progression and aggravation of mucositis during radiotherapy. Radiother. Oncol..

